# Advances in Regenerative Medicine for the Treatment of Osteonecrosis of the Jaw

**DOI:** 10.31661/gmj.v13iSP1.3676

**Published:** 2024-12-08

**Authors:** Emad Taghizadeh, Seyed Mohammad Mahdi Mirmohammadi, Arezoo Khosravi, Gholamreza Mojarab, Hossein Shahoon

**Affiliations:** ^1^ Department of Oral and Maxilofacial Surgery, Faculty of Dentistry, Shahed University,Tehran, Iran; ^2^ Department of Oral and Maxillofacial Surgery, Shahid Beheshti University of Medical Sciences, Tehran, Iran; ^3^ Department of Oral and Maxillofacial Medicine, Faculty of Dentistry, Shahed University, Tehran, Iran

**Keywords:** Osteonecrosis of The Jaw, Regenerative Medicine, Biomaterial, 3D Bioprinting, Mesenchymal Stem Cells, Platelet-rich Plasma

## Abstract

Osteonecrosis of the jaw (ONJ) is a debilitating condition characterized by progressive bone tissue necrosis, commonly linked to bisphosphonates, radiation therapy, or trauma. Traditional treatments, such as surgical debridement and conservative management, often fail to fully restore bone function, driving the need for alternative therapeutic strategies. Regenerative medicine, particularly cellular therapies and biomaterials, has emerged as a promising field in ONJ treatment. This review explores recent advancements in regenerative approaches for ONJ, with a focus on Mesenchymal stem cells (MSCs) and bioengineered scaffolds. MSCs, with their dual ability to differentiate into osteoblasts and modulate immune responses, play a crucial role in bone regeneration by both forming new bone tissue and reducing inflammation. Bioengineered scaffolds, such as hydrogels, bioactive ceramics, and nanomaterials, provide essential structural support and create a conducive environment for cellular growth and tissue repair. The combination of MSCs with these biomaterials has demonstrated a synergistic effect, significantly enhancing bone healing and regeneration. Additionally, emerging techniques such as platelet-rich plasma (PRP), platelet-rich fibrin (PRF), and bone morphogenetic proteins (BMPs) offer new avenues for improving clinical outcomes in ONJ patients. However, several challenges remain, including regulatory barriers, the need for standardized cell isolation and delivery protocols, and scalability issues for clinical application. This review further examines emerging technologies, such as 3D bioprinting and personalized medicine, which offer the potential to tailor regenerative treatments to individual patients, thereby improving both the efficacy and longevity of therapies. In conclusion, while significant progress has been made in the application of regenerative medicine for ONJ, continued research is essential to address current limitations, optimize treatment protocols, and ensure broader clinical adoption. Advances in cellular therapies and biomaterials hold transformative potential for improving therapeutic outcomes in patients with ONJ.

## Introduction

Osteonecrosis of the jaw (ONJ) is a progressive and debilitating condition that leads to bone necrosis, causing pain, infection, and the exposure of necrotic bone tissue [1][2]osteoradionecrosis, traumatic, non-traumatic, and spontaneous osteonecrosis. Antiresorptive or antiangiogenic drugs cause drug-induced osteonecrosis. The combination of medications, microbial contamination, and local trauma induces this condition. Osteoradionecrosis is a severe radiation therapy side effect that can affect people with head and neck cancer. It is described as an exposed bone area that does not heal for longer than three months after the end of radiation treatment with the absence of any indications of an original tumor, recurrence, or metastasis. Trauma (tooth extraction. Its prominence has increased due to associations with long-term bisphosphonate use, denosumab, and radiation therapy for head and neck cancers [1][3][4]osteoradionecrosis, traumatic, non-traumatic, and spontaneous osteonecrosis. Antiresorptive or antiangiogenic drugs cause drug-induced osteonecrosis. The combination of medications, microbial contamination, and local trauma induces this condition. Osteoradionecrosis is a severe radiation therapy side effect that can affect people with head and neck cancer. It is described as an exposed bone area that does not heal for longer than three months after the end of radiation treatment with the absence of any indications of an original tumor, recurrence, or metastasis. Trauma (tooth extraction. Traditional treatment approaches, such as surgical debridement, antibiotics, and conservative management, show limited efficacy in restoring full jaw function or halting disease progression [5][6]. Consequently, regenerative medicine has emerged as a promising alternative, leveraging the body’s intrinsic healing mechanisms to promote tissue repair and regeneration, offering hope for improving outcomes in ONJ patients [7].

Since conventional treatments often fail to fully restore bone function, regenerative strategies, particularly those involving [8]. Mesenchymal stem cells (MSCs) and advanced biomaterials, have attracted considerable attention for their potential to address the underlying causes of ONJ [9].

MSCs, due to their ability to differentiate into osteoblasts and suppress inflammation, have shown great potential in promoting both bone regeneration and overall tissue repair [10]. Additionally, biomaterials such as scaffolds and bioactive molecules offer structural support, enhancing the localized environment for healing and bone regeneration [11]. This review explores recent developments in regenerative therapies and assesses their potential to transform ONJ management by promoting bone regeneration and restoring jaw function

## Pathophysiology and Current Treatment of ONJ

ONJ is a severe condition characterized by the progressive necrosis of bone tissue, often precipitated by impaired blood flow. Insufficient blood supply deprives bone tissue of oxygen and nutrients, leading to necrosis, which impairs healing and leaves bone exposed [2][12].

The jawbone, especially the mandible, undergoes constant remodeling due to mastication and dental procedures, which increases its metabolic demand [13]so that it can endure mechanical loading. During food ingestion, masticatory muscles generate the required masticatory force. The magnitude of applied masticatory force has long been believed to be closely correlated with the shape of the jawbone. However, both the mechanism underlying this correlation and evidence of causation remain largely to be determined. Here, we established a novel mouse model of increased mastication in which mice were fed with a hard diet (HD. Disruption of its blood supply due to antiangiogenic medications or radiation therapy results in ischemia, hypoxia, and subsequent bone necrosis [1][8]osteoradionecrosis, traumatic, non-traumatic, and spontaneous osteonecrosis. Antiresorptive or antiangiogenic drugs cause drug-induced osteonecrosis. The combination of medications, microbial contamination, and local trauma induces this condition. Osteoradionecrosis is a severe radiation therapy side effect that can affect people with head and neck cancer. It is described as an exposed bone area that does not heal for longer than three months after the end of radiation treatment with the absence of any indications of an original tumor, recurrence, or metastasis. Trauma (tooth extraction. This vascular impairment limits the delivery of immune cells and growth factors essential for healing, creating an environment where even minor injuries fail to resolve, ultimately leading to extensive necrosis [8]. Medications-related ONJ ( MRONJ) are common cause of ONJ [14]. Bisphosphonates, a primary cause of MRONJ, work by binding to hydroxyapatite in the bone and inhibiting osteoclast activity, thereby reducing bone resorption [15]. Bisphosphonates and denosumab, by inhibiting osteoclast function and angiogenesis, play a key role in disrupting the normal bone healing processes in the jaw, leading to the development of necrotic bone and its associated complications [1][16]osteoradionecrosis, traumatic, non-traumatic, and spontaneous osteonecrosis. Antiresorptive or antiangiogenic drugs cause drug-induced osteonecrosis. The combination of medications, microbial contamination, and local trauma induces this condition. Osteoradionecrosis is a severe radiation therapy side effect that can affect people with head and neck cancer. It is described as an exposed bone area that does not heal for longer than three months after the end of radiation treatment with the absence of any indications of an original tumor, recurrence, or metastasis. Trauma (tooth extraction. Denosumab reduce bone turnover by inhibiting RANKL and osteoclastogenesis, but this suppression can create a fragile microenvironment that is vulnerable to necrosis, particularly under conditions of infection, trauma, or reduced blood supply. The molecular basis of ONJ includes the dysregulation of the RANK/RANKL/OPG pathway, which is critical for osteoclast activity [4].Patients undergoing antiangiogenic therapies, such as bevacizumab, experience inhibited neovascularization, further restricting the jawbone’s capacity for repair [17]. Inflammation exacerbates the condition, as exposed necrotic bone is often colonized by oral bacteria, triggering a persistent inflammatory response. In chronic inflammation, cytokines like tumor necrosis factor-alpha (TNF-α) and interleukin-6 (IL-6) are released, which further contribute to bone degradation. [18] This elevated inflammatory state sustains necrosis by increasing osteoclast activation, thereby interfering with bone remodeling and healing processes [18][19].

ONJ may develop after radiation therapy for head and neck cancers (osteoradionecrosis), which compromises jawbone vasculature and impairs healing [1]osteoradionecrosis, traumatic, non-traumatic, and spontaneous osteonecrosis. Antiresorptive or antiangiogenic drugs cause drug-induced osteonecrosis. The combination of medications, microbial contamination, and local trauma induces this condition. Osteoradionecrosis is a severe radiation therapy side effect that can affect people with head and neck cancer. It is described as an exposed bone area that does not heal for longer than three months after the end of radiation treatment with the absence of any indications of an original tumor, recurrence, or metastasis. Trauma (tooth extraction. Traumatic injuries, such as dental extractions, can also trigger ONJ, particularly in patients with predisposing factors like medication use or systemic conditions [12][20].

## Current Treatment and Challenges

Treating ONJ remains challenging due to its multifactorial nature, involving impaired bone remodeling, disrupted blood supply, and chronic inflammation [21]. Current treatment approaches include a combination of conservative management, medications, and surgical interventions. These strategies focus on symptom control, infection management, and minimizing disease progression, but they often fail to fully restore bone function or prevent recurrence [5][21][22].

Medications typically include antibiotics to control infection and nonsteroidal anti-inflammatory drugs (NSAIDs) or opioids for pain management [14]. Discontinuation of antiresorptive drugs like bisphosphonates may be considered to prevent further disease progression, although this is controversial, as it may exacerbate underlying conditions such as osteoporosis or metastatic bone disease [23]. Surgical interventions, such as debridement and resection, aim to remove necrotic bone and stimulate healing [24]. However, their success is limited due to the jaw's impaired healing capacity, often resulting in frequent recurrences, especially in advanced ONJ cases [25]. Conservative management, used for mild ONJ cases, includes non-invasive options like improved oral hygiene, limited surgery, and close monitoring. While these approaches help manage symptoms and delay more invasive procedures, they do not address the underlying bone necrosis, limiting their potential for complete recovery [5][26]. The primary limitation of conventional therapies lies in their symptomatic management, rather than addressing the underlying causes of ONJ, such as impaired bone remodeling and poor vascular supply [26]. Medications can control infection and alleviate pain but do not promote the regeneration of necrotic bone [23].

Surgical procedures, while sometimes necessary, are invasive and often fail to prevent recurrence, particularly in patients with compromised bone healing [24][27]. Conservative strategies, though useful in delaying invasive interventions, cannot reverse the necrotic process, resulting in persistent disease and functional impairments [26]. The limitations of existing treatment methods highlight the urgent need for innovative regenerative strategies that address the underlying causes of ONJ, such as impaired bone remodeling and inadequate vascular supply [23]. Emerging regenerative medicine approaches, such as cellular therapies and biomaterials, offer a promising pathway to enhance bone regeneration, improve vascularization, and restore jaw function more effectively than traditional methods [9][11].

## Advances in Regenerative Medicine

Regenerative medicine is revolutionizing ONJ treatment by focusing on tissue repair, regeneration, and the replacement of necrotic bone with functional, living structures [25]. Emerging cellular therapies and biomaterials are at the forefront of these developments, offering improved bone regeneration and vascularization in affected regions, overcoming the limitations of conventional treatments [28]. Biological therapies capitalize on the natural mechanisms of tissue repair, employing biologic agents that promote regeneration at the cellular and molecular levels [29]. Each therapeutic approach offers unique mechanisms of action, ranging from the stimulation of osteogenesis to modulation of the immune response, making them highly effective in the management of ONJ [23].

## Biological Therapies

**Figure-1:**
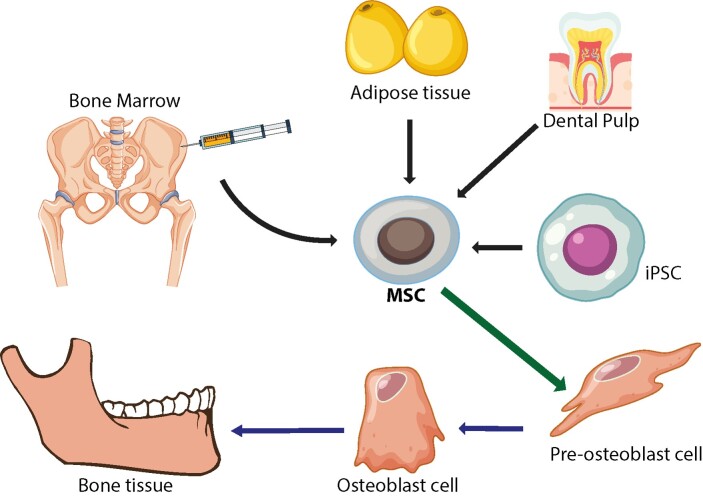
Figure 1. Schematic representation of MSC differentiation into osteoblasts for bone tissue formation. MSCs can be derived from various tissue sources, including bone marrow, adipose tissue, dental pulp, and iPSC. Once harvested, MSCs undergo differentiation into pre-osteoblast cells and further into mature osteoblasts. These osteoblast cells are responsible for forming new bone tissue, as illustrated in the diagram. The arrows represent the process of MSC differentiation and the sources from which MSCs can be obtained.

**Table:** Table1. Overview of Commonly Employed Biological Therapies in Studies for ONJ

**Therapy Type**	**Advantages**	**Limitations**	**References**
**Stem Cells**	High regenerative potential, anti-inflammatory properties, promotes soft tissue and bone repair.	Still in experimental stages; possible ethical and immune rejection concerns.	Yang, et al. [32]​ Zheng, et al. [10]
**PRP**	Improves soft tissue healing and accelerates bone regeneration.	Limited efficacy in advanced stages of ONJ and variability in preparation protocols.	Ricotta, et al.[35]
**PRF**	Autologous, reducing infection rates, and better control of inflammatory response, low cost.	Limited sample size, needs larger clinical trials. not be effective in all ONJ stages.	Mourão, et al. [36]
**BMP**	Strong bone regeneration capabilities, often used in combination with PRP for better outcomes.	High cost, off-label use for ONJ, risk of adverse effects like heterotopic bone formation.	Cicciu, et al. [33] Min, et al. [37]

Biological therapies are at the forefront of regenerative medicine for ONJ, leveraging the body's natural healing mechanisms to repair and regenerate damaged bone [28]. These therapies capitalize on the natural mechanisms of tissue repair, employing biologic agents that promote regeneration at the cellular and molecular levels [16]. Each therapeutic approach offers unique mechanisms of action, ranging from the stimulation of osteogenesis to modulation of the immune response, making them highly effective in the management of ONJ [7][10]. Key regenerative strategies include employing growth factors, stem cells, platelet concentrates, and cytokines, each designed to accelerate bone healing and repair through distinct mechanisms. For example, therapies like platelet-rich plasma (PRP) [30] and platelet-rich fibrin (PRF) deliver concentrated doses of growth factors that accelerate healing, [31] while MSCs differentiate into osteoblasts, facilitating bone regeneration [32]. Additionally, bone morphogenetic proteins (BMPs) are used to directly stimulate bone formation by activating osteogenic pathways [33]. Such innovations are proving pivotal in ONJ management, not only promoting faster recovery but also improving long-term outcomes by addressing the underlying causes of bone degeneration [34]. Table-1 below provides a comprehensive overview of the most commonly employed biological therapies in clinical studies aimed at treating osteonecrosis, highlighting their benefits and limitations.

Stem Cells

One of the most promising developments in regenerative medicine for ONJ is the application of MSCs. These multipotent stem cells can be sourced from various tissues, including bone marrow, adipose tissue, and dental pulp [34]. MSCs can also be derived from induced pluripotent stem cells (iPSCs), further expanding their therapeutic potential (Figure-1) [38]. MSCs have shown considerable promise in bone regeneration, largely due to their capacity to differentiate into osteoblasts, which are essential for new bone formation and tissue repair (Figure-1) [39].

In addition to promoting osteogenesis, MSCs exhibit immunomodulatory properties that reduce inflammation and foster an environment conducive to tissue healing [34]. Preclinical studies have shown strong evidence that MSCs improve bone repair in animal models of osteonecrosis of the jaw (ONJ) [32][40][41]. For instance, Yang et al. [32] and Matsuura et al. [41] both reported that MSC transplantation could stimulate bone regeneration and normalize key markers associated with osteonecrosis.

PRP

PRP is an autologous, blood-derived product enriched with platelets and growth factors, such as platelet-derived growth factor (PDGF), transforming growth factor-beta (TGF-β), and vascular endothelial growth factor (VEGF) [30][35]. These bioactive molecules are instrumental in wound healing processes, promoting angiogenesis (the formation of new blood vessels), and stimulating the proliferation of osteoblasts and fibroblasts, which are crucial for bone and soft tissue repair [35].

PRP, prepared by centrifuging a patient’s blood to isolate platelets and growth factors, has shown potential to enhance bone regeneration. By improving the local healing environment, PRP accelerates tissue repair and promotes bone regeneration, though its efficacy depends on consistent preparation methods [30].

Although clinical studies have yielded promising results, the efficacy of PRP can vary significantly based on its preparation methods and the concentration of growth factors [42]. This variability underscores the need for standardized protocols to ensure consistent therapeutic outcomes [43]. Several studies have shown that PRP can substantially enhance bone regeneration in ONJ by optimizing the local healing environment [30][35][44]. However, inconsistencies in PRP preparation techniques across studies highlight the importance of establishing uniform clinical protocols to improve reliability and effectiveness [45].

PRF

**Figure-2:**
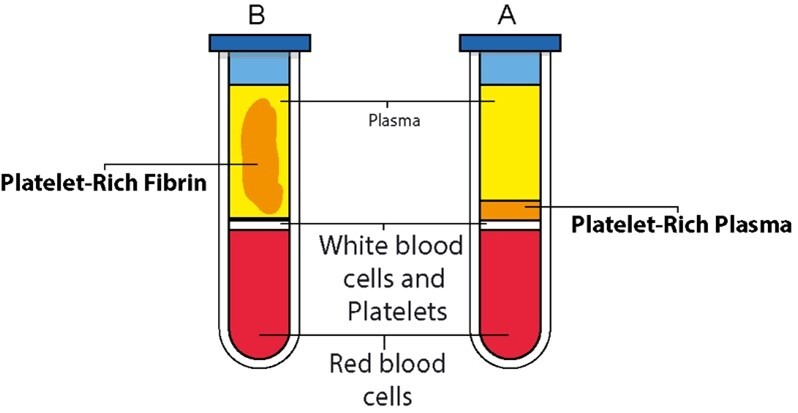
Figure 2. Comparison of PRP and PRF preparations in centrifuged blood samples. Tube A (PRP): This tube contains anticoagulated material. It depicts the separation of blood components after centrifugation to obtain Platelet-Rich Plasma (PRP). Tube B (PRF): This tube does not contain anticoagulated material. It shows the preparation of Platelet-Rich Fibrin (PRF), where a fibrin clot is formed instead of a liquid plasma component.

PRF represents a significant advancement in regenerative medicine as a second-generation platelet concentrate [36]. Unlike PRP, this method is derived from a patient's blood without the use of anticoagulants [46]. Figure-2 illustrates a comparison between PRF and advanced PRF.

During centrifugation, a fibrin matrix naturally forms, entrapping platelets, leukocytes, and a concentrated array of growth factors [47]. This fibrin scaffold gradually releases growth factors over an extended period, making PRF especially valuable for promoting sustained tissue regeneration and long-term healing processes [48]. Abo-Heikal et al. [46]conducted a clinical trial that highlighted the bone healing capabilities of PRF and its simpler preparation technique compared to PRP as a regenerative scaffold.

In the treatment of ONJ, PRF has gained increasing popularity due to its dual role in stimulating bone regeneration and facilitating wound healing [36]. The slow and steady release of growth factors from PRF helps maintain a prolonged regenerative environment, which supports not only angiogenesis (formation of new blood vessels) but also tissue repair at a cellular level [31]. Additionally, the fibrin matrix itself serves as an excellent scaffold, providing a structural foundation that encourages cell migration and the formation of new tissues [48]. This makes PRF particularly promising as an adjunct therapy, especially when combined with bone grafts or other regenerative materials to optimize clinical outcomes [36].

Furthermore, a systematic review by Muñoz-Salgado et al., [31] supports the efficacy of PRF in managing MRONJ, pointing to its enhanced regenerative properties and sustained release of growth factors as key contributors to improved clinical outcomes. PRF thus stands out as a versatile and powerful tool in ONJ treatment, providing both immediate and long-term benefits through its scaffold and slow-growth-factor-release mechanism [31][47].

BMPs

BMPs, particularly BMP-2, are powerful osteogenic agents within the transforming growth factor-beta (TGF-β) superfamily, renowned for their ability to induce MSCs differentiation into osteoblasts, which are crucial for bone formation and regeneration [49]. BMP-2 has emerged as a key player in promoting bone healing, making it a promising candidate in the treatment of ONJ [33].

Several studies have demonstrated the potential of BMP-2 in ONJ therapy. Research by Min et al., [37] illustrates that BMP-2 significantly enhances bone regeneration, particularly following surgical interventions such as sequestrectomy, where necrotic bone is removed. BMP-2 aids in accelerating the healing process by stimulating new bone formation in the affected area. Similarly, Moon et al.[49] found that BMP-2 significantly improved bone regeneration in these fractures, underscoring its potential to counteract drug-induced bone remodeling inhibition.

Despite these promising results, the clinical application of BMPs, including BMP-2, in ONJ treatment remains limited. The primary reason for this is the absence of large-scale clinical trials that would validate its efficacy and safety in diverse patient populations [49]. Additionally, concerns over the cost of BMP therapies and potential side effects, such as ectopic bone formation and inflammation, have also tempered widespread adoption in clinical settings [33][37].

## Improvements in Biomaterials

Recent advancements in biomaterials have substantially increased the potential for bone regeneration, particularly in addressing conditions [28]. By facilitating cellular activity and providing necessary structural support, these materials create an optimal environment for bone repair [7]. Breakthroughs in scaffold design, nanomaterials, and biofunctionalization are driving innovation in regenerative medicine, with a focus on promoting osteogenesis and improving tissue integration [28].

## Scaffold Design

Scaffolds, made from biocompatible materials like collagen, hydroxyapatite, and synthetic polymers, play a crucial role in bone regeneration by replicating the extracellular matrix (ECM) and providing mechanical strength alongside a porous architecture conducive to cellular growth [7]. Their design is central to the success of regenerative therapies, as they offer a three-dimensional framework that supports cell attachment, proliferation, and differentiation [50].

Materials such as hydroxyapatite and bioactive ceramics are engineered to mimic the native bone environment [11]. Hydroxyapatite, for example, closely resembles the mineral composition of bone, enhancing its osteoconductivity and integration with surrounding tissues [51]. These scaffolds do more than provide structural support they actively promote new bone formation by fostering osteoblast adhesion and activity, crucial for effective regeneration [11].

## Hydrogels

Hydrogels are another pivotal class of biomaterials, particularly well-suited for delivering cells and growth factors to necrotic regions [52]. Their high-water content and flexible structure emulate the ECM, enabling efficient encapsulation and sustained release of therapeutic agents [53]. Engineered for gradual degradation, hydrogels serve as a controlled-release system for bioactive molecules, such as BMPs and vascular endothelial growth factor (VEGF) [54]. These biomolecules are key in promoting bone regeneration and vascularization, making hydrogels indispensable in tissue engineering [53].

By creating a supportive environment for MSCs survival and function, hydrogels ensure the continued delivery of regenerative signals, making them an ideal platform for therapeutic interventions in bone repair [52].

## Nanomaterials

Nanomaterials are transforming regenerative medicine by enhancing the precision of stem cell delivery and boosting bioactivity [55]. Nanoparticles such as nanohydroxyapatite, silica-based particles, and gold nanoparticles are employed to improve targeted delivery of stem cells and growth factors [11]. By functionalizing these nanoparticles, therapeutic agents can be precisely directed to necrotic areas in ONJ, increasing treatment efficiency [9].

Moreover, nanostructured scaffolds mimic the nanoscale architecture of bone, which is essential for cell signaling and tissue development [56]. These materials interact with cells at a molecular level, promoting osteogenic differentiation and accelerating bone regeneration [9]. Nanoparticles also enable controlled release of bioactive molecules like BMPs, further enhancing the osteoinductive properties of treatments. As an example, Harikrishnan et al., [11] demonstrated that nanoengineered polycaprolactone combined with nanohydroxyapatite scaffolds exhibits strong osteogenic potential, making them ideal for repairing large bone defects.

## Biofunctionalization

Biofunctionalization involves modifying biomaterials to enhance their biological performance, particularly in osteoconductivity and tissue integration [28]. This is achieved by incorporating bioactive molecules such as growth factors and peptides into the surface of scaffolds to improve their interaction with host tissues. Functionalizing scaffolds with BMPs or VEGF, for example, can boost both osteogenesis and angiogenesis, critical factors in the successful regeneration of bone in ONJ patients [7][28].

Additionally, surface modifications like plasma treatments and chemical grafting are being employed to further improve biomaterial integration with native bone [57]. These techniques increase surface roughness and alter chemical properties, enhancing cell attachment and proliferation [28]. Furthermore, biofunctionalized scaffolds can be engineered to release therapeutic agents in response to specific biological cues, creating a more adaptive and dynamic healing process that responds to the evolving needs of the regenerating tissue [57].

## Synergistic Approaches in ONJ Treatment

The integration of cellular therapies and biomaterials provides a synergistic and comprehensive approach to ONJ treatment, significantly enhancing bone regeneration through combined biological and structural support [22]. Cellular therapies, such as MSCs, provide a biological platform for tissue repair by promoting osteogenesis and modulating the immune response [7]. On the other hand, biomaterials like scaffolds and hydrogels offer essential structural support, facilitating the survival, proliferation, and differentiation of MSCs into osteogenic cells [58]. This integration not only accelerates the healing process but also improves the quality of bone regeneration, leading to better outcomes compared to using either therapy in isolation [7].

In an animal study, Zhang et al., [52] demonstrated that the delivery of MSCs using a hyaluronic acid hydrogel (HA-Gel) combined with nanohydroxyapatite/poly-ε-caprolactone (nHP) scaffolds significantly enhanced bone repair in a rat cranial defect model. This combination promoted angiogenesis, which is vital for tissue regeneration. These findings are consistent with those reported by Yang et al., [32] who demonstrated that MSC-derived exosomes, when applied to large bone defects, enhanced vascularization and bone regeneration by stimulating endothelial cells and upregulating pro-angiogenic factors. These studies emphasize the importance of targeting angiogenesis in bone healing and the potential of MSC-loaded hydrogel scaffolds in ONJ treatment.

Bakhtiari moghadam et al., [51] further highlighted the efficacy of combining MSCs and PRP with hydroxyapatite/collagen scaffolds, demonstrating a significant improvement in bone regeneration in critical-sized defects. Razmara et al., [57] also demonstrated the effectiveness of PRP-saturated collagen scaffolds in improving bone regeneration and reducing osteonecrosis in a MRONJ rat model.

Also, an animal study demonstrated that PRP effectively prevents MRONJ following tooth extraction by promoting both soft tissue healing and bone formation [59]. These findings suggest that PRP holds significant potential for clinical application, not only in treating ONJ but also in preventing its onset [57][59].

Nanostructured scaffolds, which mimic the nanoscale architecture of bone, have shown even greater potential in enhancing MSC differentiation and accelerating bone regeneration [10].For example, Raghav et al., [9] demonstrated that these nanoscaffolds, when combined with MSCs, promoted osteoblast differentiation and facilitated faster, more comprehensive bone repair in jawbone defects.

Furthermore, Adolpho et al. [60] revealed that combining MSCs with a P(VDF-TrFE)/BaTiO3 scaffold, in conjunction with photobiomodulation therapy (PBM), significantly improved bone formation in rat calvarial defects. The integration of electrospun scaffolds, MSC injections, and PBM therapy resulted in superior bone regeneration compared to scaffold treatment alone. Zhang et al., [10] also highlighted the potential of coating poly-L-lactic acid/silk fibroin (PLLA/SF) nanofiber scaffolds with osteoblast-derived extracellular matrix (O-ECM), which significantly enhanced the osteogenic differentiation of iPSC-MSCs.

This study aligns with findings from Wu et al., [56], who demonstrated that ECM-modified scaffolds promote osteogenic differentiation and improve bone healing, evidenced by increased expression of key osteogenic markers such as Runx2, osteocalcin, and collagen type I.

These collective findings demonstrate the efficacy of combining cellular therapies, such as MSCs and PRP, with advanced biomaterials in treating ONJ [61]. This synergistic approach enhances osteogenesis, accelerates healing, and improves structural integrity, offering great promise for future ONJ treatment strategies.

## Challenges and Future Directions

Ethical Challenges

The use of MSCs in regenerative medicine offers significant potential for the treatment of ONJ, but it also raises several ethical concerns, particularly with regard to cell sourcing [34]. Adult-derived MSCs, such as those sourced from bone marrow, adipose tissue, or dental pulp, generally pose fewer ethical challenges, as they are collected from consenting adult donors or the patients themselves [62]. However, the use of iPSCs, which are created by reprogramming adult somatic cells into a pluripotent state, introduces a new set of ethical considerations.[38] While iPSCs avoid the controversy surrounding embryonic stem cells, they pose challenges related to genomic instability during reprogramming, which can lead to potential risks in clinical use [63]. Additionally, issues of informed consent and ownership of genetic material need careful consideration, especially as iPSC-based products move towards commercialization [64].

Moreover, MSCs carry risks of tumorigenesis, particularly the formation of teratomas from iPSCs if not fully differentiated before use [38]. Even MSCs, if improperly handled, can undergo genetic mutations that increase the risk of abnormal growth. Moreover, immune responses remain a concern, especially with allogeneic therapies, which may require immunosuppressive treatments to avoid rejection, further complicating their clinical use [63].

Regulatory and Standardization

A major challenge lies in navigating the regulatory landscape, particularly in gaining approvals for cellular therapies like MSCs, which require rigorous safety and efficacy validation [65]. These therapies require stringent approval from regulatory agencies. Extensive clinical trials are needed to prove both efficacy and safety, with concerns over tumorigenesis or immune reactions remaining a major hurdle [63]. The classification of cellular products and compliance with Good Manufacturing Practice (GMP) standards adds further complexity to the approval process [66]. Another critical challenge is the standardization of protocols for cell isolation, culture, and delivery, as inconsistent methods lead to varied clinical outcomes [62].

Long-term Durability and Effectiveness of Biomaterials

Biomaterials, which are crucial for supporting bone regeneration in ONJ, face their own set of challenges, particularly with large-scale production and patient-specific customization [67]. Biomaterials like scaffolds and hydrogels must meet stringent biocompatibility and bioactivity standards while maintaining the mechanical properties necessary to support bone growth.[68] Scaling up the production of these materials to meet clinical demand, without sacrificing quality, is a significant barrier. Furthermore, many ONJ cases require patient-specific solutions, especially when treating large or complex defects in the jawbone [67]. Customizing scaffolds or implants to the precise anatomical needs of individual patients, while maintaining cost-efficiency and regulatory compliance, adds another layer of complexity [67].

Future Directions

Advances in bioprinting have opened new doors for creating tailored scaffolds, but widespread implementation remains challenging due to cost and manufacturing constraints [69]. Looking forward, the future of regenerative medicine in ONJ lies in the development of more personalized treatments and the integration of cutting-edge technologies such as 3D bioprinting [70]. Personalized medicine, which tailors’ therapies to the unique genetic and biological profiles of individual patients, holds the potential to significantly improve treatment outcomes in ONJ [71]. Autologous cell therapies, where a patient’s own stem cells are harvested, expanded, and re-implanted, offer a personalized approach that minimizes the risk of immune rejection and maximizes regenerative potential [53].

3D bioprinting is another promising innovation, allowing for the precise fabrication of scaffolds that are customized to the patient’s bone defect [72]. By using bioinks composed of stem cells and biomaterials, 3D bioprinting can create complex, highly specific structures that mimic the natural bone architecture, promoting more effective tissue regeneration [73]. As the technology matures, it is expected that 3D bioprinting will be integrated into ONJ treatment to provide patient-specific scaffolds, enabling personalized regenerative solutions at scale [68].

## Conclusion

ONJ remains a challenging condition to treat due to its complex pathophysiology, which includes impaired bone healing, chronic inflammation, and disrupted vascular supply. While conventional treatments have limited success in fully restoring bone integrity, recent advances in cellular therapies and biomaterials have opened new avenues for improving patient outcomes. MSCs, with their ability to differentiate into osteoblasts and modulate the immune response, offer a powerful regenerative tool. When combined with biocompatible scaffolds, hydrogels, and other advanced biomaterials, these cellular therapies create an optimized environment for bone regeneration, significantly enhancing the healing process in ONJ.

The synergy between stem cell-based approaches and biomaterial innovations marks a promising shift in the treatment paradigm for ONJ. Scaffold technologies, particularly those incorporating bioactive ceramics and nanomaterials, are enhancing the structural support necessary for cell growth and bone formation. Moreover, the ability to deliver growth factors and stem cells in a controlled, sustained manner via hydrogels and biofunctionalized scaffolds is a significant advancement in the field. Preclinical and early clinical trials demonstrate that these combined approaches lead to improved bone regeneration and integration, highlighting the transformative potential of regenerative medicine for ONJ patients.

Despite these promising developments, there remain several challenges that require ongoing research. Standardizing protocols for stem cell isolation, culture, and delivery is essential to ensure consistent therapeutic outcomes. Additionally, addressing regulatory hurdles for the approval of advanced therapies, including stem cell treatments and bioengineered scaffolds, will be critical to moving these innovations from the lab to the clinic. There is also a need for improved understanding of the underlying mechanisms of ONJ, particularly with respect to how current medications, such as bisphosphonates, affect bone remodeling and healing. Ongoing innovations in regenerative medicine, such as 3D bioprinting and gene therapy, are poised to drive future advancements in ONJ treatment. As research in this area progresses, these personalized and targeted therapies could revolutionize the management of ONJ, offering patients more effective and durable solutions.

## Conflict of Interest

None declared.
